# Heterogeneity of Tertiary Lymphoid Structures and Plasma Cells in PDAC with and Without Lymph Node Metastasis

**DOI:** 10.3390/cancers17182949

**Published:** 2025-09-09

**Authors:** Mengfei Wang, Lizhi Zhang, Hailong Chen

**Affiliations:** 1 Department of Surgery, the First Affiliated Hospital of Dalian Medical University, Dalian 116044, China; mengfeiwang@dmu.edu.cn; 2 Department of Pathology, the First Affiliated Hospital of Dalian Medical University, Dalian 116044, China

**Keywords:** tertiary lymphoid structure, multiplexed immunofluorescence, spatial analysis, plasma cells, lymph node metastasis

## Abstract

**Background/Objectives**: TLSs are favorable PDAC prognostic biomarkers. However, the mechanisms underlying TLSs formation and their contribution to the humoral antitumor immune response remain poorly understood. **Methods**: We used mIF staining combined with AI-based pathological image analysis software to assess the heterogeneity in the distribution of TLSs, B cells, plasma cells, and tumor cells between N0 and N1/2 PDAC. Three scRNA-seq datasets and the TCGA-PAAD database were utilized to investigate the functional heterogeneity in B cells and plasma cells. **Results**: The TLS area and maturity in N0 PDAC were higher than those in N1/2 PDAC. The densities of memory B cells and germinal-center B cells in intratumoral mTLSs, as well as plasma cells in stromal imTLSs, were associated with the density of intratumoral plasma cells. Moreover, plasma cells in N0 PDAC exhibited stronger IgG antibody production than those in N1/2 PDAC. IgG^+^ tumor cells congregated within 40 μm of IgG^+^ plasma cells, forming an IgG^+^ plasma cell-related immune hotspot in N0 PDAC, which was not observed in N1/2 PDAC. The distance between IgG^+^ plasma cells and the nearest IgG^+^ tumor cells was a new prognosis biomarker. **Conclusions**: The TLS formation and development in N0 PDAC were better than those in N1/2 PDAC, and there is an IgG^+^ plasma cell-related immune hotspot in N0 PDAC. The TLS area and maturity and the distance between IgG^+^ plasma cells and the nearest IgG^+^ tumor cells could be PDAC prognostic biomarkers.

## Simple Summary

Tertiary lymphoid structures (TLSs) are associated with a favorable prognosis for pancreatic ductal adenocarcinoma (PDAC), one of the most lethal malignant tumors. However, the contribution of TLSs to antitumor responses remains incompletely understood. In this study, we used multiplex immunofluorescence (mIF) staining to quantitatively investigate the heterogeneity in TLSs by comparing N0 and N1/2 PDAC. We also explored the spatial distribution characteristics of IgG^+^ tumor cells around IgG^+^ plasma cells. According to our findings, the TLS area and maturity in N0 PDAC were higher than those in N1/2 PDAC, and plasma cells in N0 PDAC had a stronger antibody production function than those in N1/2 PDAC. Furthermore, the spatial analysis demonstrated the presence of a local immune hotspot surrounding IgG^+^ plasma cells in N0 PDAC. Notably, a shorter distance between IgG^+^ plasma cells and the nearest IgG^+^ tumor cells is associated with a better prognosis.

## 1. Introduction

Pancreatic ductal adenocarcinoma (PDAC) is one of the deadliest cancers [1], and neither the traditional treatments nor immunotherapies used in PDAC can provide reliable efficacy for patients [2,3]. Although there is a lack of stable and dependable prognostic markers, tertiary lymphoid structures (TLSs) are a hot prognostic marker for various tumors, including PDAC.

TLSs have the same cellular components and structures as those of lymph nodes (LNs) and are a portal for immune cell entry into the tumor microenvironment (TME), breaching the physical and chemical barriers created by tumor-associated fibroblasts (CAFs) [4]. TLS development is a process by which B cells differentiate and, ultimately, plasma cells are produced in the germinal center (GC), and it can be divided into three stages. Plasma cells infiltrating the tumor site share the same B cell receptor (BCR) sequence as that of plasma cells in TLSs [5]. Thus, TLSs with GCs are defined as mature TLSs (mTLSs). Both the TLS maturity and density are tumor-related prognostic markers. However, due to technology limitations, TLS evaluations are typically qualitative or semi-quantitative [5,6,7,8], which overlooks the correlation between TLSs at different spatial locations and plasma cell infiltration.

Lymphatic metastasis is a common unfavorable biomarker for patients with PDAC, and immune cells in LNs have also been demonstrated to participate in the origin and development of TLSs. However, the effect of tumor metastasis on tumor-draining lymph nodes (TDLNs) on TLSs and plasma cells in the PDAC TME remains unclear [7]. In the AJCC 8th edition staging system for pancreatic ductal adenocarcinoma (PDAC), N staging is determined by the presence and number of regional lymph node metastases: N0 indicates no regional lymph node involvement, N1 represents metastasis in 1–3 regional lymph nodes, and N2 indicates metastasis in 4 or more regional lymph nodes.

The development of multiple immunofluorescence (mIF) and AI-based pathological image analysis technology has added qualitative, quantitative, and spatial positioning information to the cells in the tumor microenvironment. Researchers can visualize the observed cell distribution characteristics by applying different software and subsequent data processing.

In this study, we focused on the heterogeneity of TLSs and plasma cells in PDAC with and without lymph node metastasis using public databases to detect the heterogeneity in B cell subsets and plasma cells in the TME of PDAC with lymphatic metastasis. We used H&E and mIF staining slides and AI-based pathological image analysis software to obtain the quantity variance and spatial distribution characteristics of B cell subsets, especially plasma cells, in the TME and LNs. Finally, based on the spatial distribution of IgG^+^ plasma cells and IgG^+^ tumor cells, we defined a 40 μm radius around IgG^+^ plasma cells as the local immune hotspot where IgG^+^ tumor cells accumulate in the TME of PDAC without lymph node metastasis. The distance between IgG^+^ plasma cells and the nearest IgG^+^ tumor cells is a new prognostic marker for PDAC [5].

## 2. Materials and Methods

### 2.1. Patient Cohort

A total of 69 patients with stages I–IV PDAC who underwent surgical resection between 2020 and 2023 from the First Affiliated Hospital of Dalian Medical University were included (ID: PJ-KS-KY-2023-297). In this cohort, 26 patients had LN metastasis, and 43 patients had no LN metastasis (Supplementary Materials, Table S1). We also collected TDLN tissues to detect the heterogeneity of LN tissue, including 9 metastatic TDLNs and 8 non-metastatic TDLNs from patients with PDAC and LN metastasis. The other 12 TDLNs were collected from patients without tumor metastasis. The clinical information of these patients is summarized in Table 1. Two experienced pathologists identified the TLSs and tumor, margin, and stromal regions in all slides.

The inclusion criteria of this study were as follows: (1) patients with primary PDAC; (2) resected TDLNs evaluated by pathologists; (3) a sufficient tissue size to perform mIF; (4) tumor, tumor margin, and stromal regions included in the PDAC tissues. The exclusion criteria were as follows: (1) patients who had received additional treatments before surgery; (2) an unassessed LN status; (3) an insufficient tissue size to perform mIF.

### 2.2. Spatial Region and TLS Classification

The H&E staining images (100×) were taken via Vectra 3.0 (Akoya Bioscience, Marlborough, MA, USA). Two pathologists used Qupath 0.5.1 to manually circle every TLS and tumor region in the H&E staining slides, and discrepancies were resolved by discussion and consensus. Then, we utilized the script to categorize the PDAC TME into the Tumor region, InnerMargin region (the inner 500 μm of the tumor margin), OuterMargin region (the outer 500 μm of the tumor margin), and Stroma region. This categorization was then extended to the TLSs. The InnerMargin and OuterMargin regions in the same slide were defined as the margin region in the following study.

### 2.3. Multiplex Immunofluorescence

Paraffin-embedded (FFPE), serial, 4 μm-thick tumor tissue slides and TDLN slides were heated at 60 °C for 2 h. We used the PANO 5-plex IHC kit (10002100100) (Panovue, Beijing, China) to conduct the mIF staining after deparaffinizing and rehydrating. We performed microwave antigen retrieval and incubated the tissues with primary and secondary antibodies, and we performed the fluorescence staining in every round. Microwave heating has both antigen retrieval and elution functions. After the last round of staining and microwave heating for the antibody elution, DAPI was used to perform the nuclear staining. The two mIF groups: Panel 1: CD20, CD27, Ki67, and CD138; Panel 2: CD27, CD138, IgG, and CK19. Finally, we used an antifade solution to mount the slides. Detailed information on the reagents and incubation conditions can be found in the Supplementary Materials, Table S2.

### 2.4. Image Acquisition for mIF Staining Slides

The mIF staining slides were scanned using Vectra 3. In the first round, all the slides were scanned at 100× *g* magnification, and then the region of interests (ROIs) were chosen in Phenochart 2.0.0.

#### 2.4.1. TME _Outside TLS

We obtained 3 ROIs from the PDAC TME (outside of TLS) at 200× *g* magnification at three spatial locations (the tumor, margin, and stromal regions) (9 ROIs/slide).

#### 2.4.2. TME _TLS

We scanned all the TLSs at 200× *g* magnification and divided them into mature TLSs (mTLSs) and immature TLSs (imTLSs) according to the Ki67 expression in Panel 1, and we also added spatial information for every TLS. Finally, we determined six TLS classifications according to the maturity status (mTLSs/imTLSs) and spatial distribution (locations: the intratumoral, marginal, and stromal regions).

#### 2.4.3. LN

We set the ROIs individually to scan the entire LN in 200× *g* magnification (Supplemental Figure S1a–c).

### 2.5. Image Analysis

We used Inform 2.4.4 to perform the primary image analysis, and we performed two panels of mIF staining on tonsil slides as a positive control to identify the positive cells of every primary antibody. We performed every fluorescence staining with the corresponding antibody of the two panels on PDAC sections to build a spectra library, and we performed mIF staining without fluorescence staining in every round to identify the autofluorescence for the subsequent analysis. For Panel 1, we trained the classifier to identify the GC and para-GC regions on mTLS and LN images and the imTLS regions on imTLS images based on the expressions of markers (CD20, CD27, Ki67, CD138) and the TLS morphology. For Panel 2, we trained the classifier to identify the tumor regions and TLSs based on the expressions of markers (CD138, CD27, IgG, CK19) and TLS morphology. We used the adaptive classifier function for cell segmentation based on the DAPI expression and that of other markers. For every marker, we chose at least 10–20 positive and negative cells to train the classifier in the phenotype function. Finally, the output files were input to R 4.3.1., and we used phenoptr 0.3.2 (Akoya Bioscience, Marlborough, MA, USA) to merge and consolidate them. Moreover, we also calculated the cell–cell distance [9].

### 2.6. Flow Cytometry-like Workflow

The export files of Inform 2.4.4 (Akoya Bioscience, Marlborough, MA, USA) were imported into FCS Express 7 version 7.20.0020 (De Novo Software, Pasadena, CA, USA). The marker expression was gated based on the fluorescence intensity in the phenotype from Inform 2.4.4. We also used the contour plot to visualize the heterogeneity in the spatial features of IgG^+^ plasma and IgG^+^ tumor cells between N0 PDAC and N1/2 PDAC.

### 2.7. scRNA-seq Data Collection and Analysis

We downloaded three datasets of raw PDAC scRNA-seq data with clinical information from the Genome Sequence Archive (GSA) (CRA001160) [10] and Gene Expression Omnibus (GEO) (GSE205013, GSE202051) [11,12] (Supplementary Materials, Table S3) (N0 = 48, N1 = 34, N2 = 11). All the analyses were employed in Seurat 4.3.0 (Satija Lab, New York, NY, USA) [13]. We selected all the B cells and plasma cells in the merged dataset, and we filtered all the cells to retain those of high quality (>500 genes, >1500 unique molecular identifiers, and <15% mitochondrial genes). After the normalization and scaling of the matrix, we selected 2000 highly variable genes, and the top 20 primary components were used to conduct the PCA. We used “Harmony” to eliminate batch effects between different datasets and specimens. Cell types were identified manually by matching Hallmark genes of B cells and plasma cells in the previous literature and CellMarker database [14,15,16,17], and uniform manifold approximation and projection (UMAP) was performed to visualize the B cell subsets (resolution = 0.7) [18].

We used monocle3 1.3.1 (Trapnell Lab, Seattle, WA, USA) to infer the cellular trajectory of the B cell subsets in PDAC, org.Hs.eg.db to perform GO and KEGG enrichment analyses on the differential expression genes (DEGs) of B cells in patients with and without LN metastasis, and ggplot2 3.5.1 [19] to visualize the cell frequency and differential gene expression in B cell subsets and plasma cells.

### 2.8. Bulk RNA seq Data Collection and Analysis

We downloaded The Cancer Genome Atlas-Pancreatic Adenocarcinoma (TCGA-PAAD) bulk RNA-seq data with clinical information from the TCGA database (N0 = 45, N1 = 120) (Supplementary Materials, Table S3). The authors provided the TLS classifiers of the TCGA-PAAD cohort, as identified in previously published research [20] (*n* = 74, TLS^−^= 127, TLS^+^ GC^−^ = 33, TLS^+^ GC^+^ = 10). We used Survival 3.5 to perform the survival analysis, CIBERSORT to obtain the frequency of 22 types of immune cells according to the expression matrix, and org.Hs.eg.db to perform the GO and KEGG enrichment analyses [21,22,23].

### 2.9. Spatial Analysis

We utilized phenoptr 0.3.2 to obtain the spatial information of every cell. Then, we calculated the distances between IgG^+^ plasma cells and IgG^+^ tumor cells, between IgG^+^ plasma cells and the nearest IgG^+^ tumor cells, and between IgG^+^ tumor cells and the nearest IgG^+^ plasma cells. We performed K-means clustering analysis to detect the spatial features of IgG^+^ tumor cells around IgG^+^ plasma cells in N0 PDAC, and we applied it to the cell counts measured in 20 consecutive 10 μm distance bins (ranging from 0 to 200 μm, k = 2).

### 2.10. Statistical Analysis

The Mann–Whitney U test was used to evaluate the statistically significant differences between the two independent groups, and the Kruskal–Wallis test was used to assess the statistically significant differences between the three independent groups. Spearman correlation analysis was used to identify the correlations between the density of the TLSs and B cells and plasma cells in the TME, the Kaplan–Meier method was used to plot the survival curves, and the log-rank test was used to compare the differences between two or more Kaplan–Meier survival curves. All the steps were performed on R 4.3.1.

## 3. Results

### 3.1. More TLSs in PDAC Without LN Metastasis than in PDAC with LN Metastasis

The clinical information on 69 PDAC patients (N0 = 43, N1/2 = 26) is presented in Table 1 (details are provided in the Supplementary Materials, Table S1). Most of the patients were in stages T1–T2 (69.6%), N0–N1 (95.7%), M0 (92.3%), and AJCC stages I–II (89.9%). Approximately half of the patients with LN metastasis had cholangiectasis (42.3%), which was worse than in those without LN metastasis (34.3%).

**Figure 1:**
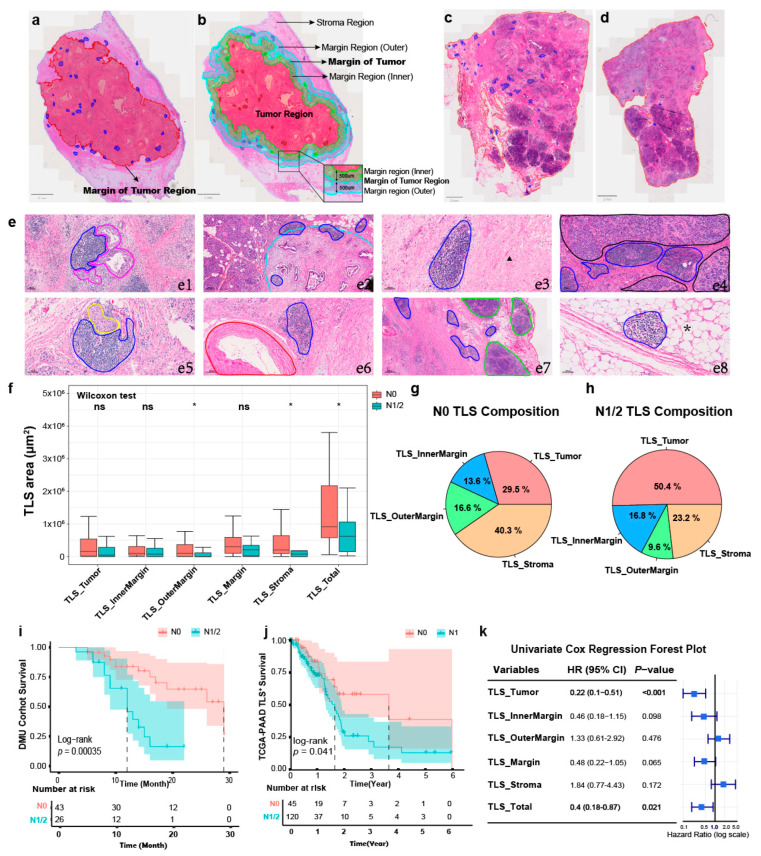
The heterogeneity of TLSs in the PDAC TME. (**a**) The pathologists drew the tumor regions (red) and TLS regions (blue). (**b**) Diagram of regions based on tumor regions (red: tumor region; green: innermargin region; cyan: outermargin region; purple: stroma region). (**c**) Distribution of TLSs (blue) in the N0 PDAC TME. (**d**) Distribution of TLSs (blue) in the N1/2 PDAC TME. (**e**) Distribution features of TLSs. (**e1**) TLS (blue) is adjacent to tumor ductal cells (pink). (**e2**) TLS (blue) is in the margin of the tumor (cyan). (**e3**) TLS (blue) is in the stroma region (black arrow). (**e4**) TLS (blue) is adjacent to the pancreas acinar (black). (**e5**) TLS (blue) is adjacent to the nerve fiber (yellow). (**e6**) TLS (blue) is adjacent to the artery (red). (**e7**) TLS (blue) is adjacent to the lymph node (green). (**e8**) TLS (blue) is in the adipose tissue (asterisk). (**f**) Heterogeneity of TLS area at different locations in PDAC with and without lymph node metastasis (Wilcoxon test, * *p* < 0.05). (**g**) Proportion of TLS in different locations in the N0 PDAC. (**h**) Proportion of TLS at different locations in the N1/2 PDAC. (**i**) Survival analysis of PDAC with and without lymph node metastasis in DMU Cohort (N0 = 43, N1/2 = 26; Log-rank test, *p* < 0.001) (**j**) Survival analysis of PDAC with and without lymph node metastasis in TCGA-PAAD Cohort (N0 = 45, N1 = 120; Log-rank test, *p* < 0.05). (**k**) Univariate COX regression analysis of TLS area at different locations.

Based on the manually drawn circle of the tumor margin and TLSs (Figure 1a), tissues were divided into four parts: the Tumor region, InnerMargin region (the inner 500 μm of the tumor margin), OuterMargin region (the outer 500 μm of the tumor margin), and Stroma region (Figure 1b). Subsequently, we also divided the TLSs into these four parts for further quantitative analysis. We summarized the common TLS locations in the PDAC TME (Figure 1e). Some TLSs were found next to tumor ductal cells (Figure 1(e1)), normal pancreatic tissues (Figure 1(e4)), nerves (Figure 1(e5)), blood vessels (Figure 1(e6)), LNs (Figure 1(e7)), and adipose tissues (Figure 1(e8)), while others could be detected in the margin (Figure 1(e2)) and stromal (Figure 1(e3)) regions of the species. There were more TLSs in patients without LN metastasis (Figure 1c) than in patients with LN metastasis (Figure 1d), the TLS areas in the OuterMargin and Stromal regions and total PDAC TME in N0 PDAC were larger than those in N1/2 PDAC (Figure 1f), and TLSs accounted for a larger proportion in the tumor regions in N0 PDAC than in N1/2 PDAC (Figure 1g,h). No LN metastasis or TLS areas in the tumor regions or total PDAC TME were favorable prognostic markers of PDAC (Figure 1i–k).

### 3.2. TLSs Were More Mature in PDAC Without LN Metastasis

**Figure 2:**
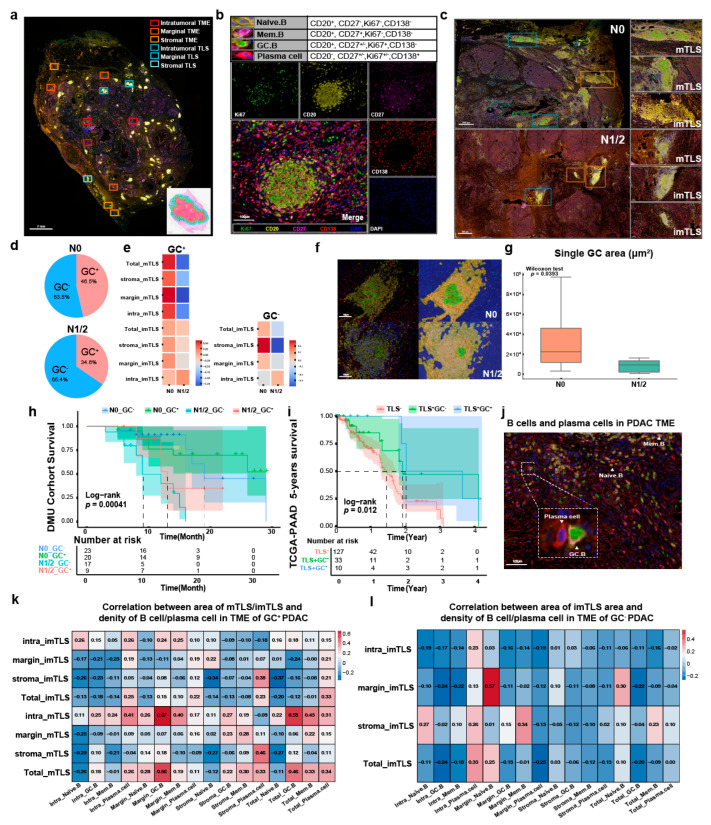
Heterogeneity of TLS maturity status. (**a**) The representative image shows the acquisition of ROIs. Localization of TME and TLS is defined based on H&E staining of serial sections. (**b**) The representative image shows mIF panel 1 staining in PDAC tissues (Ki67: green, CD20: yellow, CD27: pink, CD138: red, DAPI: blue). B cells and plasma cells are defined as Naïve.B cell (CD20^+^, CD27^−^, Ki67^−^, CD138^−^), Mem.B cell (CD20^+^, CD27^+^, Ki567^−^, CD138^−^), GC.B cell (CD20^+^, CD27^+/–^, Ki67^+^, CD138^−^), Plasma cell (CD20^−^, CD27^+/–^, Ki67^+/–^, CD138^+^). (**c**) Distribution of TLSs in the N0 and N1/2 PDAC TME (blue rectangle: mTLS; orange rectangle: imTLS). (**d**) Proportion of GC^+/–^ samples in N0 PDAC and N1/2 PDAC. (**e**) Heatmap shows the heterogeneity in the area of TLS at different locations between N0 PDAC and N1/2 PDAC in the GC^+/–^ group. (**f**) Inform 2.4.4 recognized the GC region (green), the para-GC region (yellow) and the other region (blue). (**g**) Comparison of single GC area in mTLS between N0 PDAC and N1/2 PDAC based on tissue segment by Inform 2.4.4 (Wilcoxon test, *p* < 0.05). (**h**) Survival analysis of DMU cohort between N0 GC^−^ group (*n* = 23), N0 GC^+^ group (*n* = 20), N1/2GC^−^ (*n* = 17) and N1/2GC^+^ (*n* = 9) (log-rank, *p* < 0.001). (**i**) Survival analysis of TCGA-PAAD cohort between TLS^−^ group (*n* = 127), TLS ^+^ GC^−^ group (*n* = 33) and TLS ^+^ GC^+^ group (*n* = 10) (log-rank, *p* < 0.05). (**j**) The representative image shows B cells and plasma cells in PDAC (panel 1: CD20, CD27, Ki67, CD138). (**k**) Correlation between the area of imTLS/mTLS and the densities of B cells/plasma cells at different locations of PDAC in GC^+^ samples (Spearman). (**l**) Correlation between the area of imTLS and the densities of B cells/plasma cells at different locations of PDAC in GC^−^ samples (Spearman).

We used four markers (Panel 1: CD20, CD27, Ki67, and CD138) to identify three B cell subsets and plasma cells (Naïve.B cell: CD20^+^, CD27^−^, Ki67^−^, CD138^−^; Mem.B cell: CD20^+^, CD27^+^, Ki567^−^, CD138^−^; GC.B cell: CD20^+^, CD27^+/–^, Ki67^+^, CD138^−^; Plasma cell: CD20^−^, CD27^+/–^, Ki67^+/–^, CD138^+^) [17,24,25]. The TLSs were further categorized as mTLSs/imTLSs according to the Ki67 expression. We regarded both the InnerMargin and OuterMargin regions as margin regions because of the rare mTLSs in margin regions. Channel-split images and phenotypes are presented in Figure 2b, and the heterogeneity in the TLSs between N0 PDAC and N1/2 PDAC is shown in Figure 2c. There were more TLSs in N0 PDAC, especially mTLSs.

Samples were divided into GC^+^ and GC^−^ groups according to the TLS maturity status. GC^+^ samples accounted for 46.5% in N0 PDAC and for 34.6% in N1/2 PDAC (Figure 2d). In the GC^+^ group, the mTLS area at all locations was larger in N0 PDAC than that in N1/2 PDAC (Figure 2e), and in the GC^−^ group, the stromal imTLS area was larger in N0 PDAC than that in N1/2 PDAC (Figure 2e). The single-GC area in N0 PDAC was larger than that in N1/2 PDAC (Figure 2f,g). The PDAC patients in the GC^+^ N0 group in the DMU cohort and those in the TLS^+^GC^+^ group in the TCGA-PAAD cohort had the best survival (Figure 2h,i). We identified the correlation between the mTLS/imTLS area at different locations and the density of tumor infiltration B cells and plasma cells in PDAC (Figure 2j). The intratumoral mTLS area was positively correlated with the densities of intratumoral Naïve.B (R = 0.36), Mem.B (R = 0.43), stromal Naïve.B (R = 0.31), and Mem.B (R = 0.35) cells. The intratumoral mTLS area was also positively associated with the densities of intratumoral (R = 0.26) and stromal (R = 0.28) plasma cells in the GC^+^ group (Figure 2k). In the GC^−^ group, the stromal (R = 0.34) and total imTLS (R = 0.41) areas were positively correlated with the density of intratumoral plasma cells (Figure 2l).

### 3.3. Correlation Between B Cells and Plasma Cells in TLSs and Intratumoral Plasma Cells

**Figure 3:**
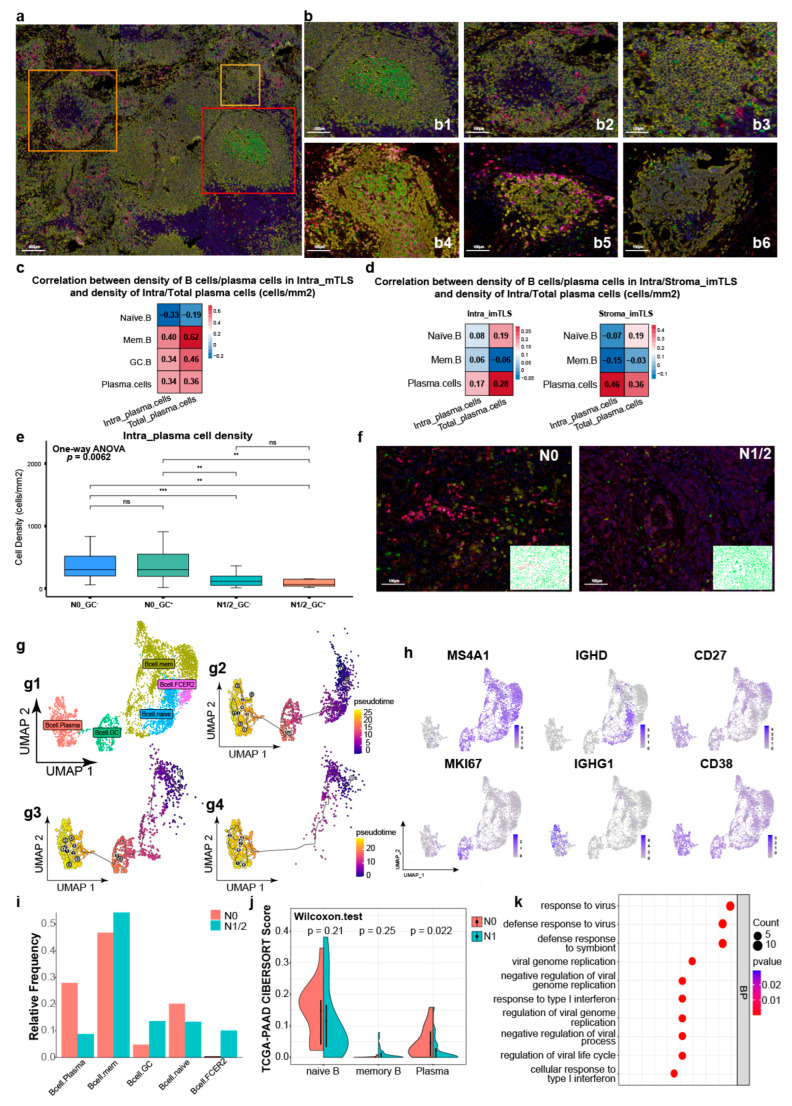
Correlation between the densities of B cells/plasma cells in mTLS/imTLS and the densities of intratumoral plasma cells. (**a**) Three classifications of lymphoid nodules in LNs (yellow: primary lymphoid nodule; orange: memory primary lymphoid nodule; red: secondary lymphoid nodule). (**b**) Three classifications of lymphoid nodules and corresponding TLSs classifications ((**b1**): secondary lymphoid nodule; (**b2**): memory primary lymphoid nodule; (**b3**): primary lymphoid nodule; (**b4**): mTLS; (**b5**): memory imTLS; (**b6**): imTLS). (**c**) Correlation between the densities of B cells/plasma cells in intra_mTLS and the densities of intratumoral/total plasma cells (Spearman). (**d**) Correlation between the densities of B cells/plasma cells in imTLS in intratumoral/stromal imTLS and the densities of intratumoral/total plasma cells (Spearman). (**e**) Comparison of the densities of intratumoral plasma cells between N0GC^−^ group (*n* = 23), N0GC^+^ group (*n* = 20), N1/2GC^−^ (*n* = 17) and N1/2GC^+^ (*n* = 9) (one-way ANOVA, *p* < 0.01; Wilcoxon test, ns: no significance; ** *p* < 0.01; *** *p* < 0.001). (**f**) Distribution of intratumoral plasma cells in N0 PDAC and N1/2 PDAC (red point: CD138^+^ plasma cells; green: CD138^−^ cells). (**g**) Definition of B cells and plasma cells based on scRNA-seq analysis in the integrated PDAC databases (N0 = 48, N1 = 34, N2 = 11). (**g1**) UMAP figure showed Naïve.B cells, Mem.B cells, GC.B cells, FCER2.B cells and plasma cells. (**g2**–**g4**) Three pseudotime differentiation pathways (white label: start point; gray label: inflection point; black point: end point). (**g2**) The first pseudotime differentiation pathway starts from Naïve.B cells to GC.B cells and finally to plasma cells. (**g3**) The second pseudotime differentiation pathway starts from Mem.B cells to GC.B cells and finally to plasma cells. (**g4**) The third pseudotime differentiation pathway starts from Mem.B cells to GC.B cells and finally to plasma cells. (**h**) Marker genes of B cell subsets and plasma cells are used to define cell types. (**i**) Relative frequency of B cells and plasma cells in N0 PDAC and N1/2 PDAC. (**j**) Comparison of Naïve.B cells, Mem.B cells and plasma cells scores between the N0 group (*n* = 45) and the N1 group (*n* = 120) based on the CIBERSORT algorithm (Wilcoxon test, *p* < 0.05). (**k**) GO-BP enrichment analysis of upregulated genes in the N1 group.

We summarized the distribution features of the B cell subsets and plasma cells in LNs (Figure 3(a,b1–b3)) and TLSs (Figure 3(b4–b6)). We detected three types of lymphoid nodules: (1) primary lymphoid nodules and imTLSs full of Naïve.B cells and scattered Mem.B cells (Figure 3(b3,b6)); (2) memory primary lymphoid nodules and memory imTLSs full of Naïve.B cells and clustered Mem.B cells (Figure 3(b2,b5)); (3) secondary lymphoid nodules and mTLSs full of Naïve.B cells, scattered Mem.B cells, and clustered GC.B cells (Figure 3(b1,b4)) [15,26]. According to previous results, the density of B cell subsets and plasma cells in intratumoral mTLSs, intratumoral imTLSs and stromal imTLSs was screened to perform the subsequent analysis.

The densities of Mem.B cells, GC.B cells, and plasma cells in intratumoral mTLSs was positively correlated with the densities of intratumoral and total plasma cells in the PDAC TME (Figure 3c). The Mem.B cell density had the strongest correlation with the total plasma cell density (Spearman, R = 0.62) (Figure 3c). The plasma cell densities in intratumoral and stromal imTLSs were positively correlated with the intratumoral plasma cell and total plasma cell densities in PDAC. The density of plasma cells in stromal imTLSs had the strongest correlation with the density of intratumoral plasma cells (Figure 3d).

We combined LN metastasis and GC information to divide the samples into four groups: N0GC^+^, N0GC^−^, N1/2GC^+^, and N1/2GC^−^. The density of intratumoral plasma cells in the N0 group was higher than that in the N1/2 group (Figure 3e). The distributions of intratumoral plasma cells in N0 PDAC and N1/2 PDAC are shown in Figure 3f.

### 3.4. Heterogeneity in B Cell and Plasma Cell Functions Between N0 PDAC and N1/2 PDAC

scRNA-seq data were used to obtain Naïve.B, Mem.B, GC.B, plasma, and FCER2.B cells from the PDAC TME (Figure 3(g1)) [26]. The marker gene expression of each subgroup is shown in Figure 3h. We used pseudotime analysis to detect the differentiation in B cell subsets and plasma cells in PDAC (Figure 3(g2–g4)). The first differentiation pathway went from Naïve.B cells to GC.B cells to plasma cells (Figure 3(g2)); the second differentiation pathway went from Mem.B cells to GC.B cells to plasma cells (Figure 3(g3)); and the third differentiation pathway went from Mem.B cells to plasma cells (Figure 3(g4)).

The percentage of plasma cells in the N0 PDAC TME was higher than that in the N1/2 PDAC TME (Figure 3i). The CIBERSORT algorithm was used to evaluate the contents of Naïve.B, Mem.B, and plasma cells in the TCGA-PAAD dataset, and it was found that the plasma cell score in the N0 PDAC TME was higher than that in the N1 group (Figure 3j). GO enrichment analysis showed that the differentially expressed genes (DEGs) upregulated in the TME of the N1 group were enriched in the type I interferon-related pathway (Figure 3k).

**Figure 4:**
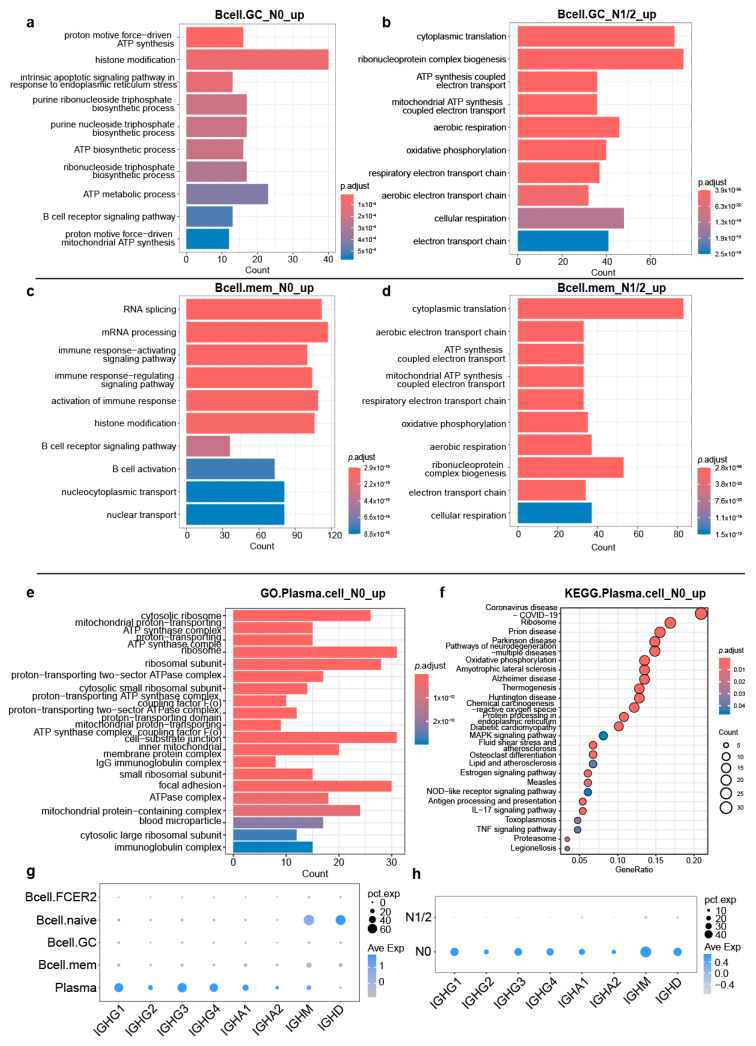
Functional heterogeneities in B cells and plasma cells between N0 PDAC and N1/2 PDAC. (**a**) GO enrichment analysis of upregulated genes in GC.B cells of N0 PDAC compared with GC.B cells of N1/2 PDAC. (**b**) GO enrichment analysis of upregulated genes in GC.B cells of N1/2 PDAC compared with GC.B cells of N0 PDAC. (**c**) GO enrichment analysis of upregulated genes in Mem.B cells of N0 PDAC compared with Mem.B cells of N1/2 PDAC. (**d**) GO enrichment analysis of upregulated genes in GC.B cells of N1/2 PDAC compared with Mem.B cells of N0 PDAC. (**e**) GO enrichment analysis of upregulated genes in plasma cells of N0 PDAC compared with plasma cells of N1/2 PDAC. (**f**) KEGG enrichment analysis of upregulated genes in plasma cells of N0 PDAC compared with plasma cells of N1/2 PDAC. (**g**) Antibody-related genes expression on B cells and plasma cells of PDAC. (**h**) Antibody-related genes expression in N0 and N1/2 PDAC.

To explore the functional differences in B cell subsets and plasma cells in the PDAC TME, we analyzed the DEGs of precursor B cell subsets (Mem.B and GC.B cells) between N0 PDAC and N1/2 PDAC. Compared with the N1/2 group, the upregulated genes of GC.B cells in the N0 group were enriched in the B cell receptor signaling pathway and ATP synthesis-related pathways (Figure 4a). Compared with the N1/2 group, the upregulated genes of Mem.B cells in the N0 group were enriched in the B cell receptor signaling pathway and B cell activation-related pathways (Figure 4c). Compared with the N0 group, the upregulated genes of GC.B and Mem.B cells in the N1/2 group were enriched in cellular respiration-related pathways (Figure 4b,d). Compared with the N1/2 group, the upregulated genes of plasma cells in the N0 group were enriched in the IgG antibody production-related pathways (Figure 4e), NOD-like receptor signaling pathway, and TNF signaling pathway (Figure 4f). The IgG antibody was highly expressed in plasma cells (Figure 4g). The expressions of IgG, IgA, IgM, and IgD antibody-related genes in N0 PDAC were higher than those in N1/2 PDAC (Figure 4h).

### 3.5. Heterogeneity in the Density of IgG^+^ Plasma Cells Between N0 PDAC and N1/2 PDAC

**Figure 5:**
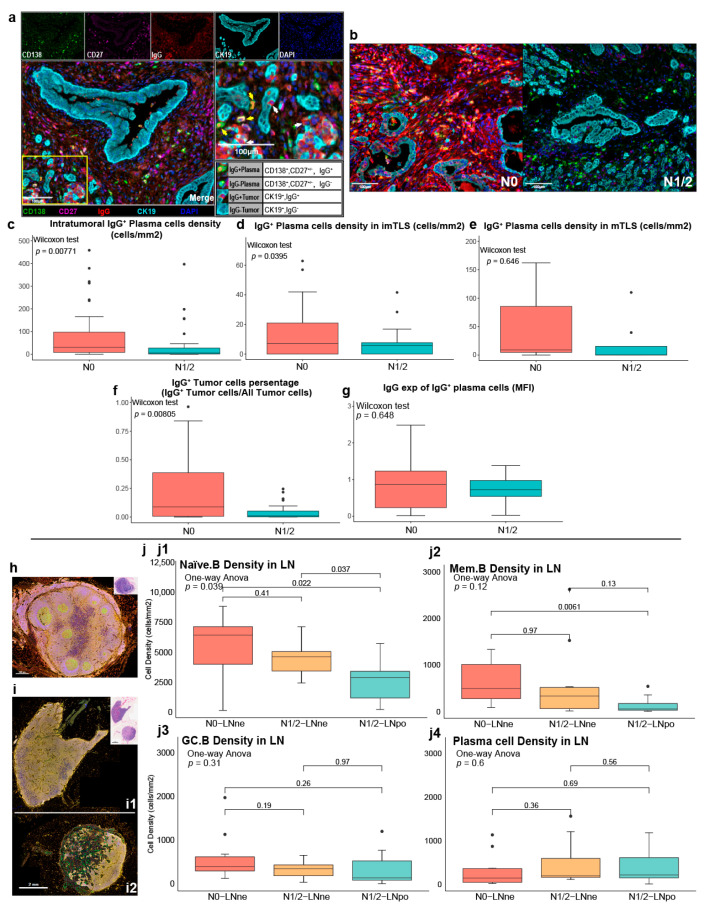
Heterogeneities in IgG^+^ plasma cells and IgG^+^ tumor cells between N0 PDAC and N1/2 PDAC. (**a**) The representative image shows distribution of IgG^+/–^ plasma cells and IgG^+/–^ tumor cells (panel 2: CD138: green, CD27: pink, IgG: red, CK19: cyan) (yellow arrow: IgG^+^ plasma cells; white arrow: IgG^+^ tumor cells). (**b**) The representative image shows IgG expression in N0 and N1/2 PDAC. (**c**) Comparison of the density of intratumoral plasma cells between N0 PDAC and N1/2 PDAC (Wilcoxon test, *p* = 0.00771). (**d**) Comparison of the density of plasma cells in imTLS between N0 PDAC and N1/2 PDAC (Wilcoxon test, *p* = 0.0395). (**e**) Comparison of the density of plasma cells in mTLS between N0 PDAC and N1/2 PDAC (Wilcoxon test, *p* = 0.646). (**f**) Comparison of IgG^+^ tumor cells percentage (IgG^+^ tumor cells num/all tumor cells num) between N0 PDAC and N1/2 PDAC (Wilcoxon test, *p* = 0.00805). (**g**) Comparison of IgG expression (MFI) of IgG^+^ plasma cells between N0 PDAC and N1/2 PDAC (Wilcoxon test, *p* = 0.648). (**h**) The representative image shows the distribution of B cells and plasma cells in N0-LNne. (**i**) Distribution of B cells and plasma cells in N1/2-LNne (**i1**) and N1/2-LNpo tissues (**i2**). (**j**) Comparison of the densities of B cells and plasma cells in N0-LNne, N1/2-LNne and N1/2-LNpo tissues. (**j1**) Comparison of the density of Naïve.B cells in N0-LNne, N1/2-LNne and N1/2-LNpo tissues (one-way ANOVA, *p* = 0.039). (**j2**) Comparison of the density of Mem.B cells in N0-LNne, N1/2-LNne and N1/2-LNpo tissues (one-way ANOVA, *p* = 0.12). (**j3**) Comparison of the density of GC. B cells in N0-LNne, N1/2-LNne and N1/2-LNpo tissues (one-way ANOVA, *p* = 0.31). (**j4**) Comparison of the density of plasma cells in N0-LNne, N1/2-LNne and N1/2-LNpo tissues (one-way ANOVA, *p* = 0.6).

Based on Panel 2 (CD27, CD138, IgG, and CK19) mIF staining, plasma cells and tumor cells were defined as IgG^+^ plasma cells (CD138^+^, CD27^+/–^, IgG^+^); IgG^−^ plasma cells (CD138^+^, CD27^+/–^, IgG^−^); IgG^+^ tumor cells (CK19^+^, IgG^+^); and IgG^−^ tumor cells (CK19^+^, IgG^−^) (Figure 5a,b). The density of intratumoral IgG^+^ plasma cells was higher in the N0 PDAC TME than that in the N1/2 PDAC TME (Figure 5c). The IgG^+^ plasma cell density within the imTLSs in N0 PDAC was higher than that in N1/2 PDAC (Figure 5d). There was no significant difference in the IgG^+^ plasma cell density within the mTLSs between N0 PDAC and N1/2 PDAC (Figure 5e). The positive percentage of IgG^+^ tumor cells in N0 PDAC was higher than that in N1/2 PDAC (Figure 5f). There was no significant difference in the expression of IgG on IgG^+^ plasma cells between N0 PDAC and N1/2 PDAC (Figure 5g), and there was no significant difference in the IgG^+^ Mem.B cell ratios of positivity (IgG^+^ Mem.B: CD138^−^CD27^−^IgG^+^/all Mem.B) between N0 PDAC and N1/2 PDAC (Supplementary Materials, Figure S2a,b).

### 3.6. Heterogeneity in B Cell Subsets and Plasma Cells in TDLNs

To clarify the LN metastasis effect on B cell subsets and plasma cells in LNs, 200× images of all the Panel 1-stained LN tissues were collected (Supplementary Materials, Figure S1a–c). The samples were divided into three groups: N0-LNne (Figure 5h), N1/2-LNne (non-metastatic lymph nodes from PDAC patients with LN metastasis) (Figure 5(i1)), and N1/2-LNpo (Figure 5(i2)). Compared with the N0-LNne group, the Naïve.B cell densities in the LN tissues of the N1/2-LNne and N1/2-LNpo groups were significantly reduced (Figure 5(j1)), and compared with the N0-LNne group, the Mem.B cell density in the N1/2-LNpo group was significantly decreased (Figure 5(j2)). There was no significant difference in the GC.B and plasma cell densities in these groups (Figure 5(j3,j4)).

### 3.7. Spatial Heterogeneity in IgG^+^ Plasma Cells and IgG^+^ Tumor Cells

**Figure 6:**
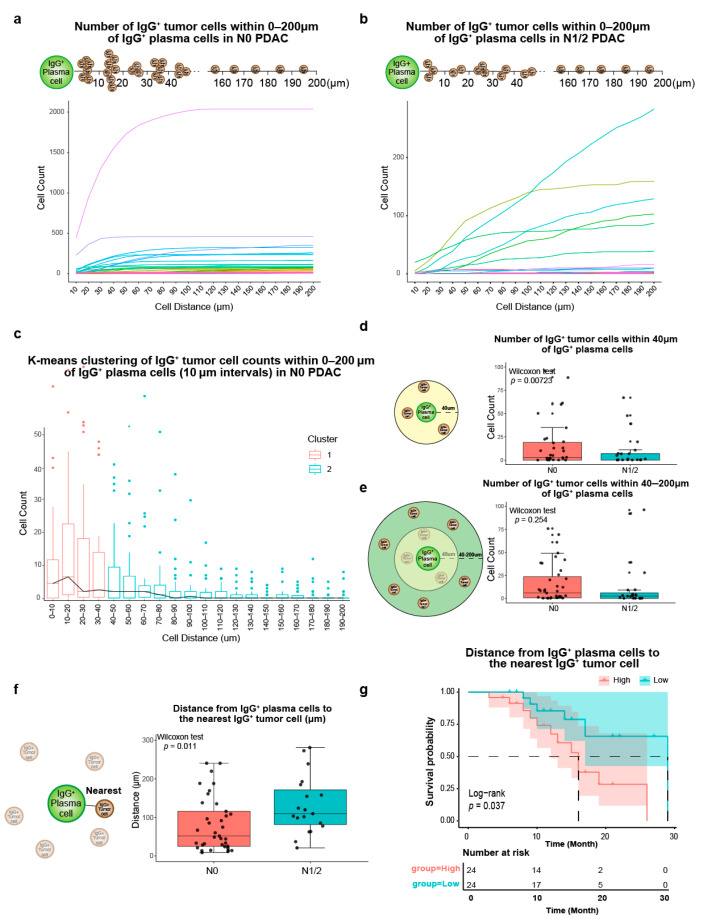
Spatial heterogeneity of IgG^+^ plasma cells and IgG^+^ tumor cells in N0 PDAC and N1/2 PDAC. (**a**) Number of IgG^+^ tumor cells within 0–200 μm of IgG^+^ plasma cells in N0 PDAC. (**b**) Number of IgG^+^ tumor cells within 0–200 μm of IgG^+^ plasma cells in N1/2 PDAC. (**c**) K-means clustering analysis of IgG^+^ tumor cell counts within 0–200 μm of IgG^+^ plasma cells (10 μm intervals) in N0 PDAC. (**d**) Comparison of the number of IgG^+^ tumor cells within 40 μm of IgG^+^ plasma cells between N0 PDAC and N1/2 PDAC (Wilcoxon test, *p* < 0.0001). (**e**) Comparison of the number of IgG^+^ tumor cells within 40–200 μm of IgG^+^ plasma cells between the N0 PDAC and N1/2 PDAC (Wilcoxon test, *p* = 0.254). (**f**) Comparison of the distance from IgG^+^ plasma cells to the nearest IgG^+^ tumor cells between N0 PDAC and N1/2 PDAC (Wilcoxon test, *p* = 0.011). (**g**) Survival analysis of distance from IgG^+^ plasma cells to the nearest IgG^+^ tumor cell (log–rank, *p* = 0.037).

The line graph of IgG^+^ tumor cells within 200 μm of IgG^+^ plasma cells shows the different distribution features between N0 PDAC and N1/2 PDAC (Figure 6a,b). There was a plateau phase starting at 100 μm in N0 PDAC, while there was no plateau phase in N1/2 PDAC. To obtain the key nodes for the change in the IgG^+^ tumor cell count per 10 μm, we used K-means clustering analysis (k = 2) to identify the turning point (Figure 6c). Cluster 1 includes the numbers of IgG^+^ tumor cells in 0–10, 10–20, 20–30, and 30–40 μm of IgG^+^ plasma cells, and Cluster 2 includes the numbers of IgG^+^ tumor cells per 10 μm in the range of 40–200 μm. The turning point was at 40 μm. The number of IgG^+^ tumor cells per 10 μm gradually decreased from 40 to 200 μm. We defined the IgG^+^ plasma extracellular 40 μm range as the “local immune hotspot” for the IgG^+^ antibody recognition of tumor cells. The number of IgG^+^ tumor cells within 40 μm around IgG^+^ plasma cells in the N0 PDAC TME was higher than that in the N1/2 PDAC TME (Figure 6d). There was no significant difference between the number of IgG^+^ tumor cells from 40 to 200 μm of IgG^+^ plasma cells (Figure 6e), and the number of IgG^+^ plasma cells within 40 μm around IgG^+^ tumor cells in the N0 PDAC TME was also higher than that in the N1/2 PDAC TME (Supplementary Materials, Figure S3a).

We obtained the nearest distances between cells via phenoptr 0.3.2 to further explore the spatial heterogeneity in IgG^+^ plasma and IgG^+^ tumor cells in the N0 and N1/2 PDAC TMEs. The distance between the IgG^+^ plasma cells and the nearest IgG^+^ tumor cells in the N0 PDAC TME was closer than that in the N1/2 PDAC TME, with a median distance of 51.6 μm (Figure 6f), and the distance between IgG^+^ tumor cells and the nearest IgG^+^ plasma cells in the N0 PDAC TME was also closer than that in the N1/2 PDAC TME (Supplementary Materials, Figure S3b). The PDAC patients with the closer distances between IgG^+^ plasma cells and the nearest IgG^+^ tumor cells had the better prognoses (Figure 6g); however, the survival analysis of the distance from IgG^+^ tumor cells to the nearest IgG^+^ plasma cells was not statistically significant (Supplementary Materials, Figure S3c).

### 3.8. Contour Plots Visualized Characteristics of Heterogeneity in Distribution of IgG^+^ Plasma Cells and IgG^+^ Tumor Cells

**Figure 7:**
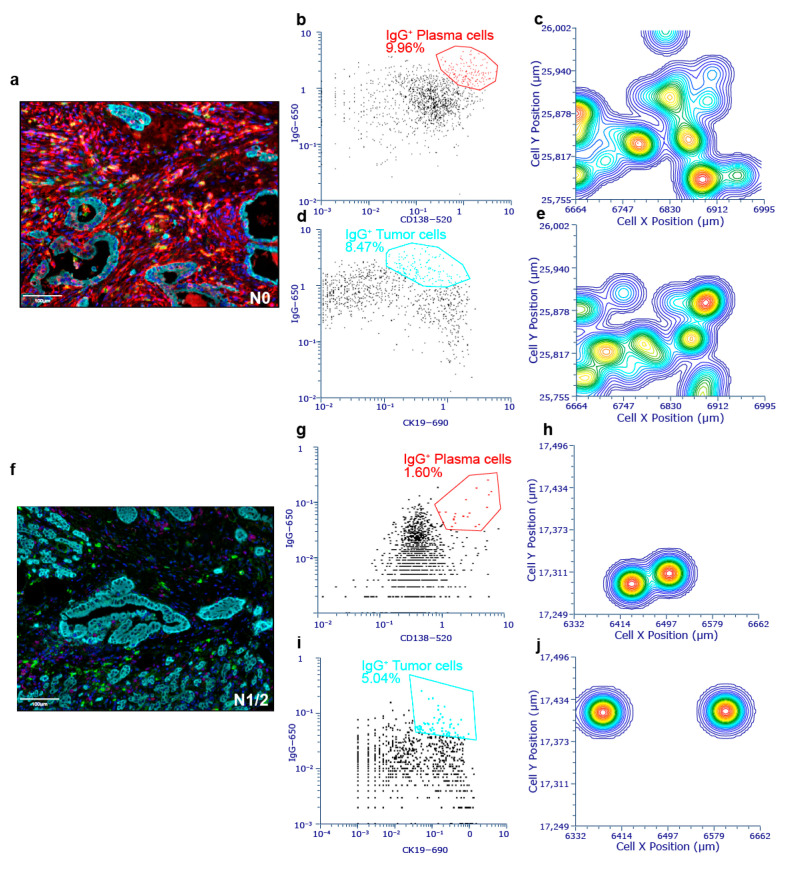
Contour plot of IgG^+^ plasma cells and IgG^+^ tumor cells in N0 PDAC and N1/2 PDAC. (**a**) The representative image showed distribution of IgG^+/–^ plasma cells and IgG^+/–^ tumor cells (panel 2: CD138, CD27, IgG, CK19) in N0 PDAC. (**b**) Tissue images are transferred to a color dot plot (red circle: IgG^+^ plasma cells). (**c**) Contour plot of IgG^+^ plasma cells in N0 PDAC (red: high density; blue: low density). (**d**) Tissue images are transferred to a color dot plot (Cyan circle: IgG^+^ tumor cells). (**e**) Contour plot of IgG^+^ tumor cells in N0 PDAC (red: high density; blue: low density). (**f**) The representative image shows distribution of IgG^+/–^ plasma cells and IgG^+/–^ tumor cells (panel 2: CD138, CD27, IgG, CK19) in N1/2 PDAC. (**g**) Tissue images are transferred to a color dot plot (red circle: IgG^+^ plasma cells). (**h**) Contour plot of IgG^+^ plasma cells in N1/2 PDAC (red: high density; blue: low density). (**i**) Tissue images are transferred to a color dot plot (cyan circle: IgG^+^ plasma cells). (**j**) Contour plot of IgG^+^ tumor cells in N1/2 PDAC (red: high density; blue: low density).

The tissue flow processing on images based on FCS Express 7 software was utilized to visualize the distribution of IgG^+^ plasma cells and IgG^+^ tumor cells in the N0 PDAC (Figure 7a) and N1/2 PDAC (Figure 7f) TME. The gates of IgG^+^ plasma cells (Figure 7b,g) and IgG^+^ tumor cells (Figure 7d,i) relied on phenotype definition data from Inform 2.4.4 software. In the N0 PDAC contour plot, multiple high–density centers of IgG^+^ plasma cells and IgG^+^ tumor cells are widely distributed (Figure 7c,e). In the N1/2 PDAC TME, there were only two high–density centers of IgG^+^ plasma cells and IgG^+^ tumor cells, and the cells were less distributed (Figure 7h,j). The number of IgG^+^ plasma cells and the percentage of IgG^+^ tumor cells in the N1/2 PDAC TME were decreased compared with those in the N0 PDAC TME.

## 4. Discussion

TLSs, as a hot biomarker, have been widely researched in a variety of tumor types [5,6,7,8,27,28]. Although intratumoral mTLSs have been demonstrated as favorable PDAC prognostic markers [29], the formation, development, and antitumor mechanisms of TLSs remain unknown [4,30,31]. Our findings confirm that the presence and maturity of TLSs are associated with improved prognosis in PDAC. Although methods to therapeutically induce TLS formation in pancreatic cancer are still under investigation, preclinical studies in other tumor models have shown that TLS-like structures can be promoted by local delivery of lymphoid chemokines such as CCL19, CCL21, and CXCL13 [32], or by activation of the lymphotoxin β receptor pathway [2]. Immunostimulatory approaches, including CD40 agonists [33] and STING agonists [34] have also been reported to foster TLS development. Moreover, immune checkpoint blockade has been associated with TLS induction in melanoma and lung cancer, although its relevance to PDAC remains to be determined [35]. Taken together, these strategies suggest that therapeutic induction or maturation of TLSs, alongside promoting effective plasma cell positioning, may provide a novel avenue to enhance antitumor immunity in PDAC.

With the development of scRNA-seq, mIF, and AI-based pathological image analysis, TLSs and immune cells in the TME can be assessed based on qualitative, quantitative, and spatial analyses. According to previous studies, LNs are an important source of immune cell initiation in TLSs [2,36]. First, we detected the heterogeneity in TLSs in the TMEs of PDAC with and without LN metastasis. Then, we obtained the correlation between the heterogeneity in TLSs and the heterogeneity in B cell subsets and plasma cells in the TME. Finally, we identified the IgG^+^ plasma cell-related immune hotspot based on spatial analysis. We demonstrated that the distance between IgG^+^ plasma cells and the nearest IgG^+^ tumor cells was a favorable prognostic marker.

Most of the previous studies on TLSs were qualitative [5,37] or semi-quantitative [6]. Based on these methods, the bias against intratumoral mTLSs is inevitable. In this study, we detected the TLS area in the tumor region, and the total TLS area was a favorable PDAC prognostic marker, emphasizing the importance of imTLSs and mTLSs at all locations in the TME. The heterogeneities in the TLS densities and maturity statuses between N0 PDAC and N1/2 PDAC cleared the LN metastasis effect in TLS formation and development, which may be caused by the decreasing density of Naïve.B and Mem.B cells in LNs. Even the negative TDLNs of LN metastasis PDAC could be influenced. Subsequently, we detected the correlation between the mTLS/imTLS areas and the B cell subset and plasma cell densities at all locations in the TME, and we extended the results to the cell densities in TLSs. To the best of our knowledge, this is the first demonstration of a positive correlation between the density of Mem.B, GC.B, and plasma cells in mTLSs and the density of intratumoral and total plasma cells in the TME. Moreover, the positive correlation between the density of plasma cells in imTLSs and the density of intratumoral and total plasma cells in the TME also confirms the importance of imTLSs. Some GC.B cells (CD20^+^Ki67^+^) were outside the GC in the TME (Figure 2j), for which there are two possible sources: (1) development from Mem.B cells in the TME [15,26]; (2) migration from TDLNs or mTLSs [26]. More mechanistic research is needed to clarify the function and development of these GC.B cells.

In this study, we identified a new type of imTLS full of Mem.B cells, which we named the memory imTLS (Figure 3(b5)), the same as the memory lymphoid nodule (Figure 3(b2)) [15]. However, the number of memories imTLSs was too small for us to set it as an independent type of imTLS. When re-encountered with antigens, Mem.B cells could rapidly differentiate into plasma cells [15]. The B cell activation pathways of Mem.B cells were upregulated in N0 PDAC, which suggests that the function of the Mem.B cells in N1/2 PDAC was impaired or inhibited. Although there was no correlation between the Mem.B cell density in imTLSs and the plasma cell density in the TME, the strong correlation between the density of Mem.B cells in mTLSs and the density of intratumoral and total plasma cells emphasizes the critical role of Mem.B cells in mTLSs and imTLSs [38]. Our pseudotime analysis suggested two potential differentiation routes toward plasma cells: a direct route from Mem.B cells to Plasma cells and an indirect route from Mem.B cells to GC.B cells and finally to plasma cells. This is in line with previous studies showing that Mem.B cells can rapidly differentiate into plasma cells during recall responses, or alternatively re-enter germinal centers for further affinity maturation before becoming plasma cells [39,40]. The transcriptional regulators reported in the literature, such as BCL6 and AICDA for the GC program, and IRF4, PRDM1 (BLIMP1), and XBP1 for plasma cell commitment, provide a mechanistic framework that is consistent with the differentiation patterns we observed. External cues, including CD40–NF-κB and IL-21–STAT3 signaling, are also known to promote plasma cell fate decisions. Although our current data cannot dissect these pathways in detail, our spatial findings suggest that patients enriched for the direct route from Mem.B cells to plasma cells may generate plasma cells more efficiently in proximity to tumor cells.

Although the functions and migration of plasma cells in TLSs remain unclear, previous studies have demonstrated that plasma cells in tumor regions have identical BCR sequences to those in TLSs [28]. Plasma cells migrate along the fibroblasts based on the CXCR4-CXCL12 axis [28]. In this study, we focused on the heterogeneities in intratumoral IgG^+^ plasma cells between N0 PDAC and N1/2 PDAC to identify the key factors affecting prognosis. Compared with N1/2 PDAC, the IgG immunoglobulin complex pathway was upregulated on plasma cells in N0 PDAC, which was confirmed via quantitative analysis based on Panel 2 staining. Finally, the heterogeneity highlights the differences in the IgG antibody production functions of plasma cells.

Plasma cells in tumor regions can kill tumor cells through antibody-dependent cell-mediated cytotoxicity (ADCC), antibody-dependent phagocytosis (ADP), and complement-dependent cytotoxicity (CDC) [17,28,41] after antibodies recognize and bind tumor cells. The general belief in previous studies was that the closer the plasma cells are to the tumor cells, the better the antitumor effect [42]. To the best of our knowledge, this is the first demonstration of the spatial features of IgG^+^ plasma cells and IgG^+^ tumor cells in the PDAC TME. According to the spatial distribution characteristics and K-means clustering analysis, we identified an IgG^+^ plasma cell-related immune hotspot (0–40 μm around IgG^+^ plasma cells) in N0 PDAC. In the following study, we also determined that a shorter distance between IgG^+^ plasma cells and the nearest IgG^+^ tumor cells is associated with a better prognosis. This study provides methodological support for spatial distance studies of the TME in other tumor types.

Although TLS density, maturity, and plasma cell immunogenicity may not directly translate into survival benefits in our current cohort, they provide important insight into the immune contexture of PDAC. TLS features can serve as predictive markers for patient stratification in immunotherapy, as mature TLS have been associated with improved responses to immune checkpoint inhibitors and other immunomodulatory strategies in several tumor types. In addition, our findings suggest that enhancing TLS formation or plasma cell functionality may represent a novel therapeutic avenue to improve antitumor immunity in PDAC. Future studies and clinical trials are warranted to explore whether modulation of TLS or humoral immune responses can ultimately improve treatment efficacy and patient outcomes.

We acknowledge that our study focused primarily on B cells and plasma cells within TLS. However, the T-cell compartment is also critical for TLS function. Previous studies have shown that mTLSs are more likely to contain organized T-cell zones with abundant CD4^+^ and CD8^+^ T cells, which cooperate with B cells to sustain antitumor immunity [2]. In contrast, imTLSs often lack such compartmentalization and may even harbor higher proportions of regulatory T cells, which could suppress effective immune responses and contribute to the impaired plasma cell function that we observed in imTLSs. Although we did not quantify T-cell subsets in the present study, these findings from the literature support the view that the balance between cytotoxic T cells and Tregs within TLS may be an important determinant of their antitumor efficacy in PDAC. Future investigations should address this interplay in greater detail.

Although this study is innovative, it still has certain limitations. First, the samples were collected at a single institution, and the sample size was insufficient. Second, we did not directly determine the antigenic specificity of the IgG produced by intratumoral plasma cells. In principle, these antibodies may target tumor-associated antigens such as MUC1 or mesothelin, patient-specific neoantigens, or even self-antigens, as reported in other cancers [43]. Nevertheless, our spatial analysis showed that IgG^+^ plasma cells and IgG^+^ tumor cells form local immune hotspots within 40 µm, suggesting that at least a fraction of the antibodies recognize tumor cell antigens in situ. Future studies combining single-cell BCR sequencing with antigen discovery platforms will be required to elucidate the precise targets of these antibodies in PDAC. Moreover, it is necessary to define the important role of the spatial features of IgG^+^ plasma cells in immunotherapies. Evidence from multiple tumor types suggests that TLS-derived plasma cells can produce IgG targeting tumor-associated antigens, such as MUC1 and mesothelin, and these antibodies may mediate antitumor effects through antibody-dependent cellular cytotoxicity, complement activation, or by facilitating antigen presentation [43]. In our study, the spatial colocalization of IgG^+^ plasma cells with IgG^+^ tumor cells within 40 µm supports the notion that at least a fraction of these antibodies recognize tumor cell antigens in situ. While we did not directly evaluate antibody specificity or effector functions, future work integrating single-cell BCR sequencing and functional assays will be required to establish the antitumor activity of TLS-derived IgG in PDAC.

## 5. Conclusions

In summary, we determined that the maturity and density of the TLSs in the TME of PDAC without LN metastasis were higher than those in PDAC with LN metastasis. B cells and plasma cells in imTLSs and mTLSs are associated with the infiltration of plasma cells in PDAC, and plasma cells in N0 PDAC have a stronger IgG antibody production function than those in N1/2 PDAC. IgG^+^ tumor cells gathered within 40 μm of IgG^+^ plasma cells, forming an immune hotspot in N0 PDAC, and the distance from IgG^+^ plasma cells to the nearest IgG^+^ tumor cells was prognostic marker for PDAC.

## Figures and Tables

**Table 1:** The clinical information of 69 PDAC patients.

Variable	N0	N1/2
Age		
<60	14 (32.6%)	6 (23.1%)
≥60	29 (67.4%)	20 (76.9%)
Sex		
Male	21 (48.3%)	18 (69.2%)
Female	22 (51.1%)	8 (30.8%)
pT stage		
T1–T2	29 (67.4%)	19 (73.1%)
T3–T4	14 (32.6%)	7 (26.9%)
pN stage		
N0	43 (100%)	0
N1	0	23 (88.5%)
N2	0	3 (11.5%)
pM stage		
M0	41 (95.3%)	23 (88.5%)
M1	2 (4.7%)	3 (11.5%)
AJCC stage		
I–II	40 (93.0%)	22 (84.6%)
III–IV	3 (7.0%)	4 (15.4%)
Cholangiectasis		
Yes	15 (34.9%)	11 (42.3%)
No	28 (65.1%)	15 (57.7%)
Operation		
Pancreatoduodenectomy	21 (48.9%)	24 (92.3%)
Distal pancreatectomy	22 (51.1%)	2 (7.7%)
TLS maturation status		
Mature	20 (46.5)	9 (34.6)
Immature	23 (53.5)	17 (65.4)

## Data Availability

The authors declare that the main data supporting the findings of this study are available within the article and Supplementary Information. The data presented in this study are available upon request from the corresponding author.

## Supplementary Materials

The following supporting information can be downloaded at: https://www.mdpi.com/article/10.3390/cancers17182949/s1, Figure S1: ROIs of the entire TDLNs tissue; Figure S2: Intratumoral IgG^+^ Mem.B cells in PDAC; Figure S3: Spatial heterogeneity of IgG^+^ plasma cells around IgG^+^ tumor cells between N0 PDAC and N1/2 PDAC. Table S1: Clinical information of DMU cohort. Table S2: Details of antibodies used in this study. Table S3: TDLNs information of databases.

## Abbreviations

The following abbreviations are used in this manuscript:

ADCC	Antibody-dependent cell-mediated cytotoxicity
FFPE	Formalin-fixed paraffin-embedding
GC	Germinal center
HR	Hazard ratio
imTLS	Immature TLS
KM	Kaplan–Meier
mTLS	Mature TLS
Mem.B	Memory B cells
mIF	Multiplex immunofluorescence
Naïve.B	Naïve B cells
PAAD	Pancreatic Adenocarcinoma
ROI	Region of interest
TLS	Tertiary lymphoid structure
TBST	Tris-buffered saline tween-20
TCGA	The Cancer Genome Atlas
TDLN	Tumor draining lymph node
TME	Tumor microenvironment
