# Supplementary material for: Heterogeneity of Tertiary Lymphoid Structures and Plasma Cells in PDAC with and Without Lymph Node Metastasis
Source: Cancers (Basel). 2025 Sep 9;17(18):2949. doi: 10.3390/cancers17182949 (PMC12468369; doi:10.3390/cancers17182949)
Supplement: Supplementary file 1 [file cancers-17-02949-s001.zip › cancers-3796281 supplement figure. xml.pdf]

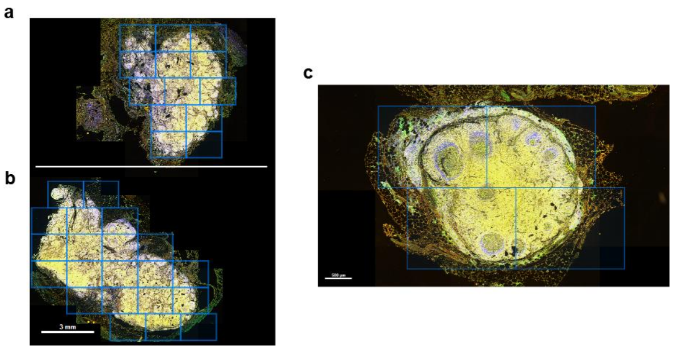

Supplementary Figure S1. ROIs of the entire TDLN tissue. (a) ROIs of the entire TDLN tissue without tumor metastasis from patients with LN metastasis. (b) ROIs of the entire TDLN tissue with tumor metastasis. (c) ROIs of the entire TDLN tissue without tumor metastasis (blue rectangle: ROI regions).

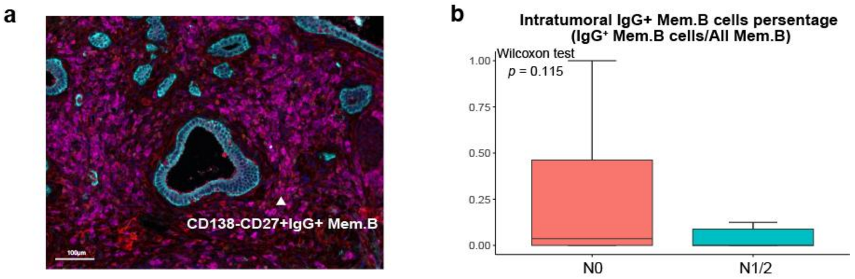

Supplementary Figure S2. Intratumoral IgG+ Mem.B cells in PDAC. (a) The representative image showed the distribution of IgG+ Mem.B cells (Mem.B: CD138-CD27-IgG+). (b) Comparison of IgG+ Mem.B cells percentage between N0 PDAC and N1/2 PDAC (Wilcoxon test, p = 0.115).

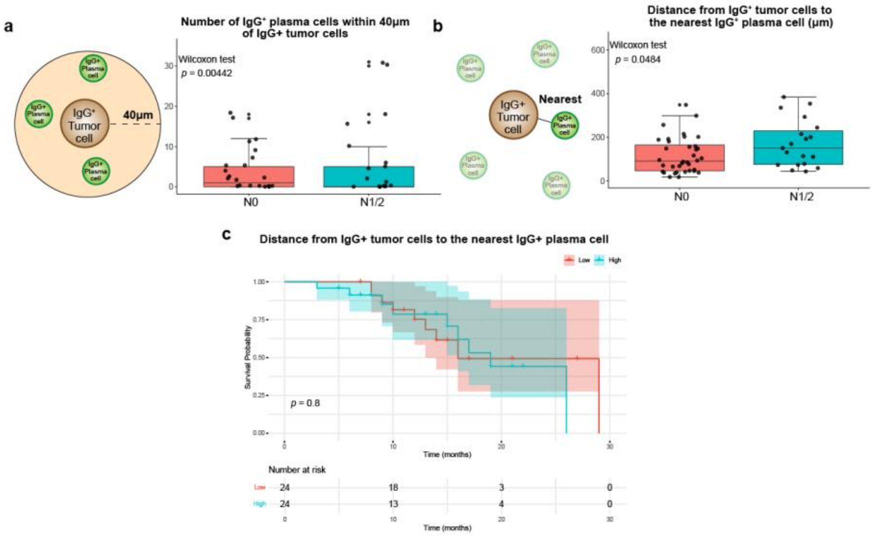

Supplementary Figure S3. Spatial heterogeneity of IgG+ plasma cells around IgG+ tumor cells in N0 PDAC and N1/2 PDAC. (a) Comparison of the number of IgG+ plasma cells within 40 μm of IgG+ tumor cells between N0 PDAC and N1/2 PDAC (Wilcoxon test, p = 0.00442). (b) Comparison of the distance from IgG+ tumor cells to the nearest IgG+ plasma cell between N0 PDAC and N1/2 PDAC (Wilcoxon test, p = 0.0484). (c) Survival analysis of distance from IgG+ tumor cells to the nearest IgG+ plasma cell (log-rank, p = 0.8) (shadows: 95% confidence intervals).
